# Diffusion of small molecules into medaka embryos improved by electroporation

**DOI:** 10.1186/1472-6750-13-53

**Published:** 2013-07-01

**Authors:** Gerlinde Jung, Markus Hug, Christian Halter, Andrea Friesenhengst, Johann Walzer, Thomas Czerny

**Affiliations:** 1 Department for Applied Life Sciences, University of Applied Sciences, FH Campus Wien, Helmut-Qualtinger-Gasse 2, A-1030, Vienna, Austria; 2 Department for Engineering, University of Applied Sciences, FH Campus Wien, Favoritenstrasse 226, A-1100, Vienna, Austria

**Keywords:** Medaka, Small molecules, Diffusion, Toxicology, Electroporation, LiCl

## Abstract

**Background:**

Diffusion of small molecules into fish embryos is essential for many experimental procedures in developmental biology and toxicology. Since we observed a weak uptake of lithium into medaka eggs we started a detailed analysis of its diffusion properties using small fluorescent molecules.

**Results:**

Contrary to our expectations, not the rigid outer chorion but instead membrane systems surrounding the embryo/yolk turned out to be the limiting factor for diffusion into medaka eggs. The consequence is a bi-phasic uptake of small molecules first reaching the pervitelline space with a diffusion half-time in the range of a few minutes. This is followed by a slow second phase (half-time in the range of several hours) during which accumulation in the embryo/yolk takes place. Treatment with detergents improved the uptake, but strongly affected the internal distribution of the molecules. Testing electroporation we could establish conditions to overcome the diffusion barrier. Applying this method to lithium chloride we observed anterior truncations in medaka embryos in agreement with its proposed activation of Wnt signalling.

**Conclusions:**

The diffusion of small molecules into medaka embryos is slow, caused by membrane systems underneath the chorion. These results have important implications for pharmacologic/toxicologic techniques like the fish embryo test, which therefore require extended incubation times in order to reach sufficient concentrations in the embryos.

## Background

Japanese medaka (*Oryzias latipes*) are small egg-laying freshwater fish that are native to brackish waters and rice paddies in South-East Asia. Economic husbandry, high fecundity, and ex utero development make them a popular vertebrate model organism in developmental biology and molecular genetics and their transparent chorion facilitates non-invasive observation. Furthermore, they are very hardy and highly resistant to common fish diseases (reviewed in [1]). These properties make medaka ideal for testing of toxic substances [2-5].

Toxicity tests are conducted to evaluate the adverse effects of chemicals or biological substances on organisms and are mainly performed by animal experiments. Even if testing of cosmetic and personal care products has been reduced over the last years, there remain numerous chemicals, such as pharmaceuticals or food additives, where animal tests are necessary. On the other hand, several programs like the OECD HPV (High Production Volume) Program or the European Union REACH (Registration, Evaluation, Authorization and Restriction of Chemicals) initiative were introduced to regulate the chemical industry globally (reviewed in [6]). They require extensive test regimes for most chemicals. Without further alternatives, these new regulations would therefore dramatically increase the number of animal experiments, particularly for mammals and birds.

Historically, fish toxicity testing plays an important role in ecotoxicology and aquatic toxicology. It is however a controversial question, whether adult fish can be a replacement for mammals and birds, because they may experience the same levels of pain and distress. One alternative is the use of embryos [7,8]. In the fish embryo toxicity (FET) test, newly fertilized eggs are exposed to a chemical for 48 hours and various lethal and sub-lethal endpoints are recorded as described by Lammer and colleagues [9]. Due to their high fecundity and the synchronous extra-uterine development of the transparent embryos, fish are particularly well suited for such a strategy. Most FET tests have been conducted with zebrafish but inter-species comparison with fathead minnow and medaka demonstrated applicability also for other species [10]. Furthermore, fish embryos are suitable for adaption to high throughput protocols [11].

Contrary to zebrafish, medaka embryonic development is relatively long, thus providing extended time for testing. In combination with their hardiness, medaka embryos therefore represent an ideal system for toxicologic/pharmacologic testing. During FET tests the substances have to diffuse into the developing embryo. Medaka embryos are surrounded by an acellular envelope, the chorion, which protects the developing embryo from environmental influences. Once fertilized, the chorion turns into a rigid structure [12] and might therefore act as an effective barrier that protects the embryo also against chemicals.

Compared to zebrafish, we found a substantially reduced sensitivity for chemicals like lithium chloride for medaka. Closer inspection revealed a limiting diffusion rate of membranes positioned closely to the embryo. Contrary to our expectations, the rigid outer chorion is readily passed by small molecules. In an effort to overcome these problems, we tested the addition of detergents. This effectively improved diffusion, but also affected the internal distribution of the chemicals. Electroporation also enhanced the uptake of fluorescent tracer molecules. When applied to Wnt-signalling, electroporation facilitated the transfer of the GSK-3 inhibitor lithium, thereby inducing deficiencies in medaka anterior-posterior development, similar to those reported for zebrafish [13].

## Methods

### Fish stocks and maintenance

Japanese medaka from the wild-type cab strain were used for this study. Adult fish were maintained at 26°C with an artificial 14 hours light and 10 hours dark cycle. Stages were determined according to Iwamatsu [14].

### Dechorionation using hatching enzyme

For dechorionation, the embryos were placed in a small drop of water on a plastic surface. Excess liquid was removed and hatching enzyme was added (10 μl hatching enzyme per 15 embryos). The embryos were incubated at 27°C until holes appeared in the chorion. The remaining chorion was removed using forceps and the embryos were kept in ERM (17 mM NaCl; 0.4 mM KCl; 0.27 mM CaCl_2_; 0.65 mM MgSO_4_) in dishes coated with agarose.

Hatching enzyme was prepared as follows: embryos with visible hatching glands were homogenized in pre-cooled PBS (0.75 μl PBS per embryo), incubated over night at 4°C and debris was removed by centrifugation (15.000 rpm, 4°C, 10 minutes). The hatching enzyme solution was stored in aliquots at −20°C.

### Diffusion experiments

Sodium fluorescein (Roth; 10 mg/ml), rhodamine B (Roth; 10 ng/ml) and acridine orange (Roth; 1 μg/ml) solutions were prepared in 1x Yamamoto’s medium (0.128 M NaCl; 0.27 mM KCl; 0.14 mM CaCl_2_; 0.24 mM NaHCO_3_). Four embryos were incubated in 50 μl staining solution for 40 minutes (if not mentioned otherwise) at 27°C protected from light. Standard post incubation washes were performed with 0.5 ml ERM at room temperature on a shaker. The embryos were washed 3 times with fresh ERM in the first seven minutes and subsequently 10, 20, 30 and 60 minutes after the incubation.

For yolk injections, sodium fluorescein (10 mg/ml) was injected into the yolk using a microinjector (Eppendorf) and borosilicate glass capillaries (Clark Electromedical Instruments).

For methylene blue diffusion, 0.001% methylene blue in 1x ERM was used. The embryos were incubated for 10 minutes at 27°C. Standard washing steps were performed as described above.

### Quantification of fluorescence intensity

Embryos were examined *in vivo* by fluorescence microscopy using a Nikon Eclipse TS100 inverted microscope equipped with an Infinity 2 camera. Pictures with defined exposure times were taken and the fluorescence intensity was quantified from the pictures with the ImageJ software [15]. The quantification of internal concentrations was performed by measuring the fluorescence intensity at different time points of washing and extrapolating back to time zero (start of washing).

As a reference we used empty cellulose sulphate beads with an average diameter of 730 μm [16], which were soaked with different concentrations of the fluorescent dyes for several days to reach equilibration. The beads were shortly washed and then transferred into mineral oil, where they were immediately quantified as described above.

### Electroporation

Electroporation of medaka embryos was performed in 1× Yamamoto’s medium. Prior to electroporation the embryos were pre-incubated with the staining solution for 40 minutes in Gene Pulser® disposable Cuvettes with 0.4 cm gap width (Bio-Rad) at 27°C and then electroporated in the same cuvettes. For better comparison, the control experiments were also performed in cuvettes omitting the electroporation pulses. Electroporation was performed with a home-made device consisting of a function generator (sine wave with added DC-voltage and variable modulation depth), a pulse generator (generating a defined number of pulses with varying length), a switch (regulated by the pulse generator, controlling the output of the function generator to the amplifier) and an amplifier connected to the cuvette filled with 100 μl 1× Yamamoto’s medium. An oscilloscope was used to display the signals. The conditions for the experiments were: modulation frequency 1 to 10^5^ Hz, modulation depth 1; voltage 5 to 50 V; 1 to 3 pulses with intervals of varying length.

For the experiments mimicking chorion diffusion alone, dead embryos were prepared as follows: fifty embryos at stage 17 were electroporated in 400 μl 1× Yamamoto’s medium using 3 pulses of 60 ms with 200 ms interval, 60 V, 0.5 A and 35 kHz, followed by 30 minutes incubation in 1× ERM at 27°C.

### Lithium chloride

Embryos at stage 14 were incubated in 100 μl 0.4 M lithium chloride (Roth) solution for 10 minutes at 27°C, followed by electroporation using the following settings: modulation frequency 330 Hz; voltage 15 V; pulse time 100 ms; 1 pulse. After diffusion and electroporation the embryos were washed as described before. Embryos were examined *in vivo* by light microscopy using a Zeiss Stemi 2000-C microscope equipped with an AxioCam HRc camera.

## Results

We previously tested the effects of the Wnt signalling pathway on medaka anterior-posterior development by overexpressing *wnt1*[17]. Another well-established way to activate this pathway is the application of the GSK-3 inhibitor lithium [18]. However, when we used conditions established for zebrafish embryos [13] no effects could be observed. Except at very high concentrations (above 1 M) where a low number of embryos showed phenotypes. We therefore reasoned that the diffusion through the chorion into the embryos was not efficient. In order to understand this problem in more detail and to find possible solutions, we started analysing the diffusion properties of the embryo’s chorion and membranes. As small and easily quantifiable molecular tracers we selected fluorescing substances.

### Diffusion into medaka embryos

We compared several fluorescing small molecules. Depending on their molecular properties, they were effective at variable concentrations and differed in their distribution within the egg (embryo/yolk). We finally selected fluorescein, which showed enrichment within the embryo (see below) and therefore appeared well suited as a model for small molecules affecting embryonic development. To quantify the uptake, we compared fluorescence spectroscopic determination of egg extracts and quantification of live embryos under a fluorescence microscope (measurement of pixel intensity of microscopic pictures, for details see Materials and Methods). Although the spectroscopic measurements were more sensitive, we selected the microscopic determination of fluorescence intensity as a standard quantification method. This allowed us to follow the same individuals during different stages of development and thereby to qualify tissue distribution, phenotypic alterations and survival of the embryos.

**Figure 1:**
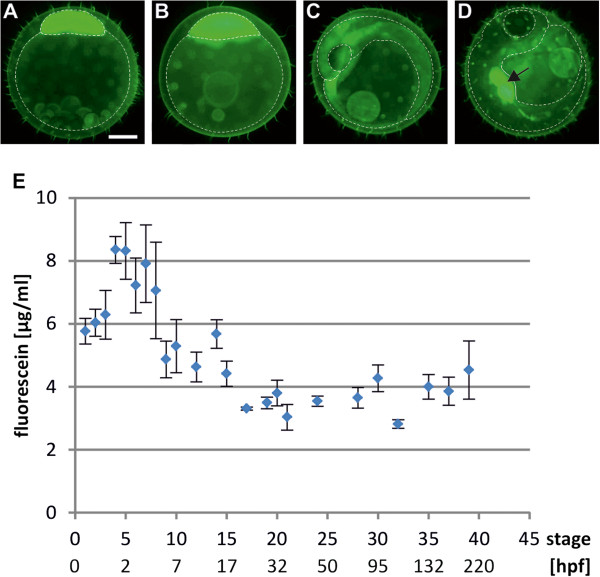
**Diffusion of the small molecule fluorescein into the medaka embryo.** Embryos at various stages were incubated with 10 mg/ml fluorescein for 40 minutes at 27°C. Pictures were taken after 30 minutes of washing and the fluorescence intensity was measured. The dotted lines demarcate the outlines of: (**A**,**B**) yolk and blastoderm, and (**C**,**D**) yolk and embryo. Embryos are shown in lateral view. Black arrow indicates the liver and gall bladder. (**A**-**D**) Distribution of fluorescein uptake during four representative embryonic stages: **A**, stage 2a; **B**, stage 8; **C**, stage 26; **D**, stage 38. The internal concentration of fluorescein (average distribution in embryo/yolk assumed) for different embryonic stages is shown in (**E**). Scale bar 250 μM. Abbreviations: hpf, hours post fertilization.

To determine the optimal conditions, the embryos were incubated in the staining solution at 27°C with variable concentrations of fluorescein for different durations. Best quantifiable results were obtained after 40 min incubation with 10 mg/ml fluorescein and 30 min washing time. Even at considerable higher concentrations (100 mg/ml), fluorescein did not cause any phenotypic alterations in the embryos. These conditions allowed sensitive quantification of the internalized dye over a wide linear range and the settings were therefore applied to all further diffusion experiments. We first analysed the uptake of fluorescein during medaka embryonic development. At the 1-cell stage the internalized fluorescein was localized almost exclusively in the zygote (Figure 1A) and remained in the proliferating cells during early development (Figure 1B). Also later, fluorescence was detected preferentially in the embryo (Figure 1C). For incubation shortly before hatching the signal became enriched in the embryonic liver (Figure 1D). Measurement of the pixel intensity showed a relatively constant fluorescein uptake throughout all developmental stages, except from the 4-cell stage to early morula where the signal slightly increased (Figure 1E).

We also looked at the fate of fluorescein. For this, embryos at the 1-cell stage were treated as described before, but the fluorescence signal was determined after 4 days of incubation in ERM. This extensive washing removed the majority of fluorescein. However, residual fluorescence was detected in the gallbladder and the liver. Even in bright field pictures (Additional file 1) yellow staining of fluorescein can be seen in the gallbladder. Therefore, the fate of fluorescein in older medaka embryos is comparable to that of mammals [19].

### Diffusion barriers within the medaka egg

**Figure 2:**
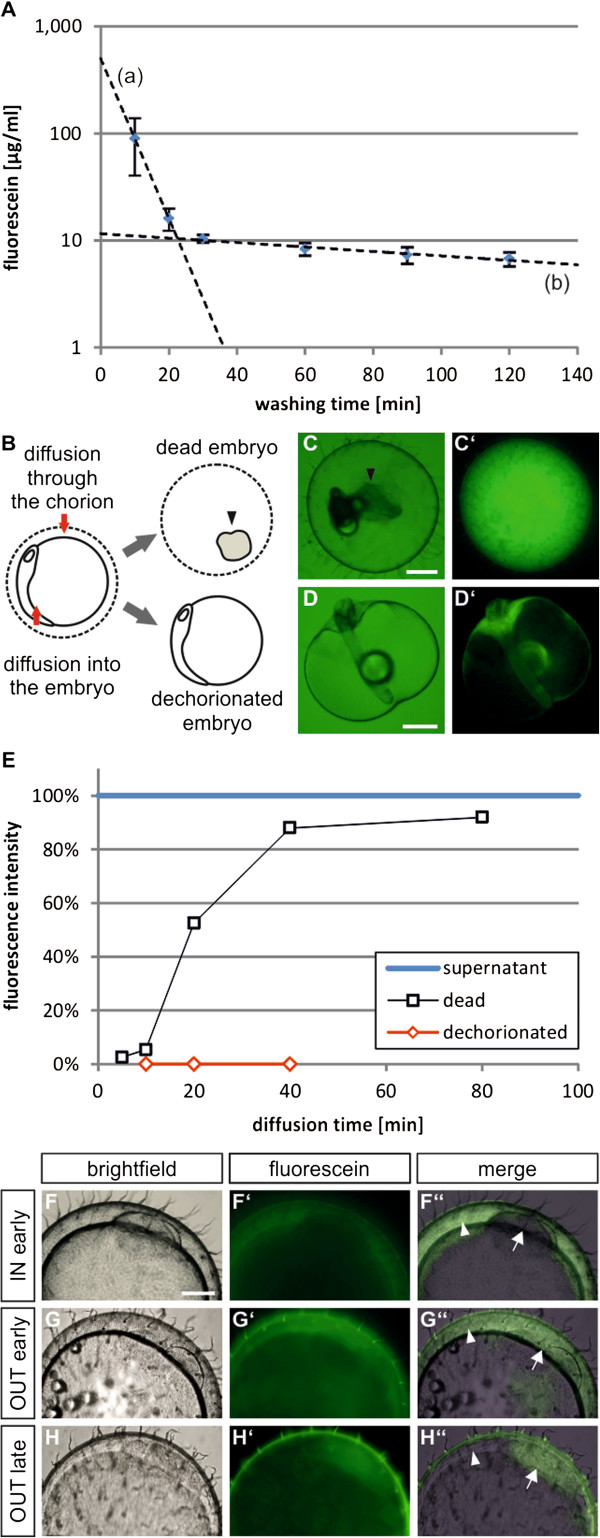
**Diffusion barriers within the medaka egg.** Stage 17 embryos were incubated with 10 mg/ml fluorescein for 40 minutes at 27°C and internal concentrations were determined at different time points of washing (**A**; mean values of 8–10 eggs are shown +/− SEM). (**A**) Dotted lines indicate the bi-phasic out-diffusion: (**a**) fast loss of the signal (first 2 measuring points), (**b**) slower second phase (best-fit of last 4 measuring points). (**B**, left side) shows a schematic view of an egg and the consecutive diffusion steps (red arrows). (**C**) Bright field picture of a dead embryo, representing diffusion through the chorion; the black arrow head indicates the shrunken embryo/yolk with the surrounding membranes. (**D**) Dechorionated embryo at stage 22 in dorsal view with anterior being at the top, (**C**’, **D**’) corresponding fluorescent images. (**E**) Concentrations of fluorescein in dead or dechorionated embryos after different incubation times. The concentration of the supernatant (blue) was set to 100%. Several measuring points (7, 20 and 30 min) were taken and the initial concentration at the onset of the washing calculated by extrapolation (compare **A**). (**F**-**H**) Eggs in brightfield, fluorescence and merged pictures. (**F**-**F”**) Distribution of fluorescein after 10 minutes of in-diffusion (IN early) and 2 minutes of washing (1 mg/ml fluorescein). (**G**-**G”**) Out-diffusion after 40 minutes of incubation (10 mg/ml fluorescein) and 5 minutes washing (OUT early) and (**H**-**H”**) same conditions as in (**G**) but 30 minutes of washing time. Scale bar 250 μM.

Overall, the amount of internalized fluorescein was relatively low. After 40 minutes of in-diffusion between 3 and 9 μg/ml (uniform distribution assumed) were detected in the embryo which represents less than 0.1% of the external concentration (10 mg/ml). Due to the high concentration in the supernatant the internal fluorescein concentration could not be quantitated during in-diffusion. In order to learn more about the kinetics we therefore analysed the out-diffusion. Several fast washing steps directly after the incubation were necessary to remove excess material from the surface of the eggs. First reproducible results were obtained after 7 min of washing (including a 3-fold exchange of the supernatant). Additional measuring points after 20, 30, 60, 90 and 120 minutes revealed two phases for diffusion: a fast loss of signal during the first 30 minutes, followed by a slower second phase (Figure 2A). Calculation of the diffusion half-times for fluorescein resulted in 4 minutes for the first, and 2.4 hours for the second phase.

Based on the bi-phasic out-diffusion observed for fluorescein, we questioned which structures of the medaka egg are responsible for the diffusion kinetics. The egg is shielded by a rigid acellular envelope, the chorion, which represents a prime candidate for a diffusion barrier [3]. However, inside the chorion the embryo and the yolk are covered by additional extra-embryonic membrane systems, which could also affect diffusion. In order to differentiate between these possibilities we manipulated the eggs. In eggs with dead embryos the yolk and membranes shrink to a small ball within the chorion (Figure 2B,C). The quantified fluorescence signal in the egg therefore mainly depends on the diffusion through the chorion. In order to qualify the diffusion properties of the membrane systems covering embryo and yolk, we used hatching enzyme to dechorionate the eggs (Figure 2B,D; for experimental details see Methods).

Quite unexpectedly, diffusion of fluorescein into dead embryos resulted in strong fluorescence (Figure 2C’), indicating that the chorion is readily passed by small molecules. Quantification of the signals (Figure 2E, for details see Methods) showed that the interior concentration almost reached saturation after 40 minutes (50% after 20 minutes). On the other hand, diffusion into dechorionated embryos was extremely slow, resulting in an average concentration similar to that for intact eggs (<0.1%) after 40 minutes (some of the dechorionated embryos showed high signals, which was however due to disruption of the fragile membranes). Therefore, not the chorion, but membrane systems within the medaka egg represent the main diffusion barrier for small molecules. These data would predict that diffusion of small molecules into intact embryos should rapidly proceed through the chorion into the perivitelline space, but would then be stopped at inner membranes. To test this hypothesis also with life embryos, we looked at eggs shortly exposed to fluorescein (10 minutes), which should be sufficient to reach high signal levels in the perivitelline space, but not in the embryo. Indeed, after a short washing step (2 minutes) the expected distribution was visible (Figure 2F-F”). Based on the previous experiments (Figure 1), the cells of the embryo should however show a signal when exposed for longer times (40 minutes). After a short washing step fluorescein should therefore be detectable in both the perivitelline space and the embryo (Figure 2G-G”; white arrowhead and arrow, respectively). During extended washing fluorescein should rapidly disappear from the perivitelline space (fast out-diffusion), but a signal would be retained in the embryo (slow out-diffusion). The expected results could be observed (Figure 2H-H”). In order to verify this model also with another small molecule we tested methylene blue. As seen for fluorescein, methylene blue rapidly (after 10 minutes of incubation) accumulated in the perivitelline space (Additional file 2A). Longer washing times (20 minutes) quickly removed methylene blue again, but no signals were detectable in the cells of the embryo (Additional file 2B).

An internal diffusion barrier is also in perfect agreement with the bi-phasic out-diffusion profile (Figure 2A). The first phase represents loss of fluorescein from the perivitelline space (line a) and the second phase (line b) is caused by the considerably slower diffusion out of the embryo/yolk (resulting in a low steady state concentration in the perivitelline space). The half-time calculated for the initial out-diffusion (4 minutes) roughly fits to that of in-diffusion through the chorion (20 minutes; a factor of 5 differing the values can be explained by the larger interior volume of dead embryos compared to that of the perivitelline space). Therefore, the slow diffusion of small molecules into medaka embryos is caused by membrane systems close to the embryo and dechorionation does not improve the uptake.

### Detergents affect membrane behaviour

In an attempt to overcome this diffusion barrier, we first focussed on the injection of the small molecules. Injection into single cells of the embryo is well established for early stages of development, leading to a rapid distribution in all cells of the embryo. During later development injection into large internal cavities, like the neural tube, would be an option. However this is again limited to certain stages. In order to accomplish uniform diffusion into the embryos, we tested injection into the yolk, a method that is often performed in zebrafish. Within 30 minutes the injected fluorescein evenly distributed throughout the yolk (Additional file 3A-C and F-G). However, the detection of signals in the embryo was only possible after 4 hours (Additional file 3D,K) and the obtained concentrations were low. Furthermore, yolk injection in medaka results in low reproducibility, due to rapid clogging of the injection needles by the sticky yolk.

**Figure 3:**
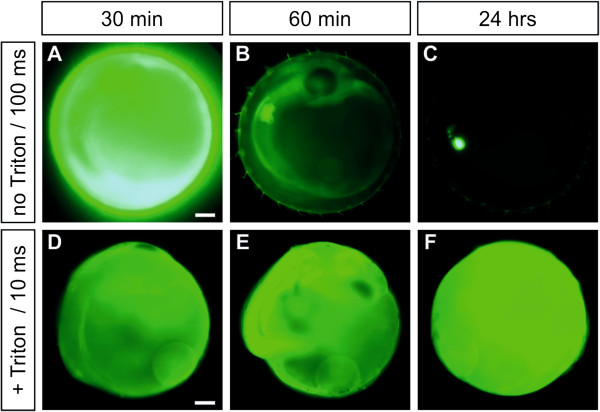
**Incubation with detergents.** 3 day old embryos incubated for 40 minutes with fluorescein (10 mg/ml) at different time points of washing without addition (**A**-**C**; no Triton), or together with 0.03% Triton X-100 (**D**-**F**; + Triton). Embryos are shown in lateral view, anterior to the top. Note that pictures (**A**-**C**) were taken with 100 ms exposure time, whereas (**D**-**F**) with 10 ms. The accumulation of fluorescein in the liver/gallbladder at later stages can be seen in (**C**). Scale bar 250 μM.

Next we tested incubation of the embryos with detergents. Contrary to ionic detergents like SDS, which appeared highly toxic, Triton X-100 worked well. The embryos tolerated concentrations up to 0.01% (40 minutes incubation) without showing phenotypes. At 0.03% Triton X-100, the survival was reduced to 69% and for 0.1% Triton to 25%, but no obvious phenotypes were detectable in the surviving embryos. Co-incubation with 0.03% Triton X-100 increased the fluorescein uptake of the eggs by a factor of 6 compared to control embryos treated with ERM (Figure 3A,D; 30 minutes of washing; note that different exposure times were used for the pictures). Interestingly, extended washing did not reduce the intensity of the fluorescent signal of detergent treated embryos (Figure 3D-F), indicating that the dye became trapped in the embryo/yolk. The signal intensity therefore increased to 20 fold after 60 minutes and to more than 80 fold after 24 hours (Figure 3B,E and C,F) compared to the control embryos at the same time points. Furthermore, Triton-treated eggs showed signals throughout the embryo and the yolk (Figure 3D-F), whereas fluorescein was clearly enriched in the embryos of non-treated eggs (Figure 3B and Figure 1A-D). Therefore, addition of Triton X-100 improves the uptake of small molecules into the egg, but also affects their distribution between compartments, making the assessment of pharmacologic/toxicologic properties difficult.

### Electroporation improves small molecule uptake into embryos

**Figure 4:**
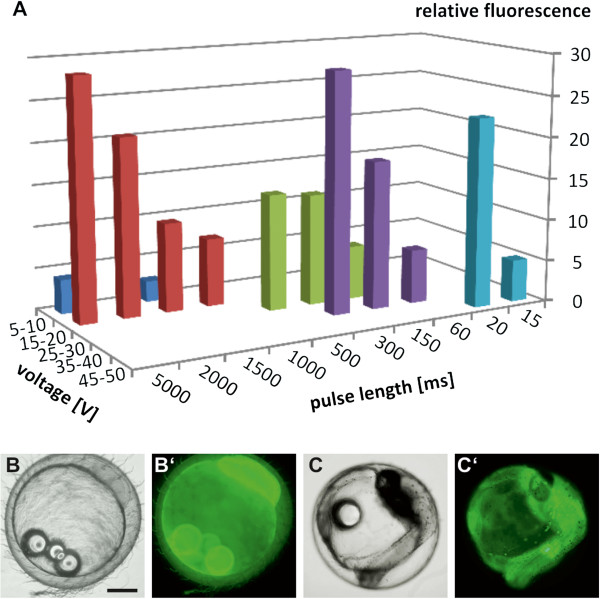
**Optimization of electroporation conditions.** (**A**) Embryos at stage 17 were incubated with 10 μg/ml fluorescein for 40 minutes at 27°C. Subsequent electroporation was performed at 35 kHz with varying voltages and pulse lengths. Relative fluorescence indicates fold quantified signal compared to that of non-electroporated, but otherwise equally treated reference embryos. Similar results were obtained for early (**B**, **B’**, stage 4) and late (**C**, **C’**, stage 31) embryos. Embryos are shown in lateral (**B**,**B’**) and dorso-lateral (**C**,**C’**) view. Scale bar 250 μM.

Another method for enhanced transport across membranes is electroporation. Hostetler and colleagues used direct current-shifted radio frequency pulses to generate transgenic medaka fish [20]. We used the same conditions as a starting point (modulation frequency, 35 kHz; voltage, 25 V; pulse duration, 10 ms; using 3 pulses with 1 second pulse interval) and measured the fluorescence intensity of fluorescein in electroporated stage 17 embryos. Due to the fact that fluorescein readily passes the chorion, we incubated the eggs prior to electroporation for 40 minutes with the staining solution. As a result high concentrations of the dye accumulated in the perivitelline space (compare Figure 2F”). To overcome the rate limiting membrane systems we applied electric pulses varying the voltage and pulse duration over a wide range. Finally, we obtained optimal fluorescence intensity and survival at 15 V and 5 seconds pulse duration (Figure 4A and Additional file 4). In contrast to Hostetler and colleagues, these conditions represent a single pulse of extended length, but with comparable low voltage. Additional pulses did not improve fluorescein uptake into the embryos. Nevertheless, other combinations, such as high voltage with short pulses also resulted in significantly higher fluorescence intensity than diffusion alone. However, the combination of long pulses with high voltage was impossible to apply due to a reduced survival rate. The same conditions were also applicable for younger (Figure 4B’) and older embryos (Figure 4C’).

**Figure 5:**
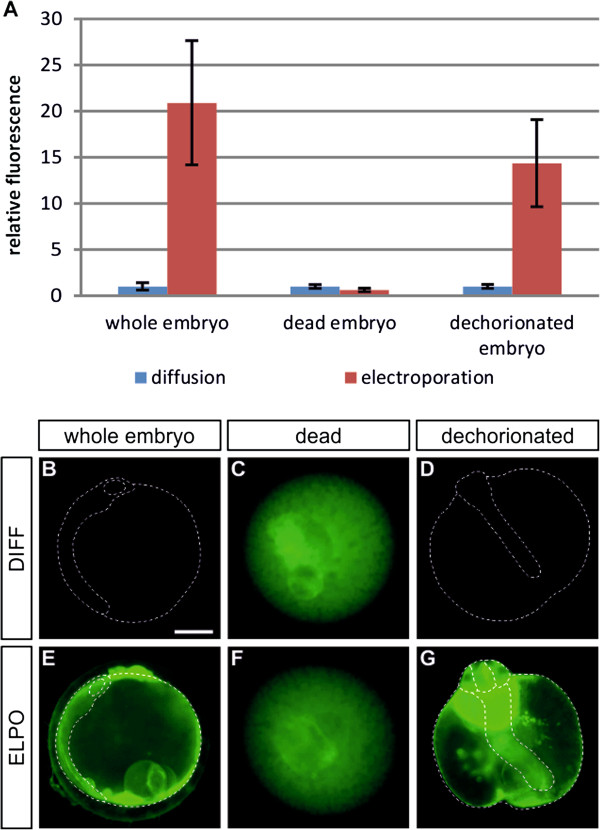
**Electroporation affects the transfer of fluorescein through inner membranes**, **but not the chorion.** Embryos were incubated with 10 μg/ml fluorescein for 40 minutes (**A**, blue bars; **B**-**D**); subsequent electroporation (**A**, red bars; **E**-**G**) was performed using 35 kHz, 15 V, 5000 ms, 1 pulse. Pictures were taken after 30 minutes washing time. Embryos are shown in lateral (**B**,**E**) and dorsal (**D**,**G**) view with anterior to the top. Scale bar 250 μM. Abbreviations: ELPO, electroporation; DIFF, diffusion.

In order to verify that electroporation improves the rate limiting diffusion through inner membrane systems, we performed the same experiments with dead and dechorionated embryos (Figure 5). Electroporation did not change the signal intensity of fluorescein in dead embryos, indicating that it has no effect on the uptake through the chorion (Figure 5A,C,F), whereas dechorionated embryos showed a nearly 15-fold increase of dye intensity after electroporation (Figure 5A,D,G). Therefore, similar to whole embryos (Figure 5A,B,E), the dechorionated embryos still contain membranes acting as diffusion barriers, which can be made permeable by electroporation.

**Figure 6:**
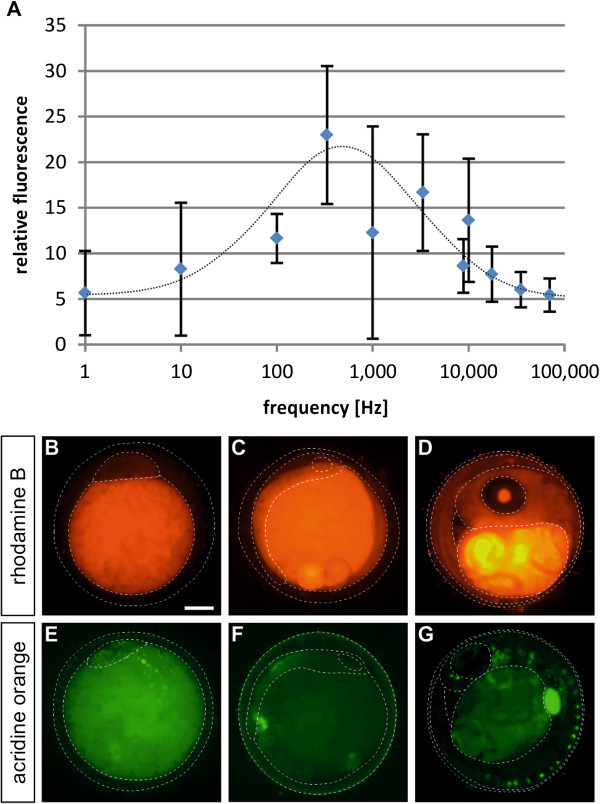
**Electroporation of rhodamine B and acridine orange.** (**A**) Optimisation of the modulation frequency of electroporation. The electroporation was performed at 15 V; 5000 ms; 1 pulse using variable frequencies. Relative fluorescence indicates fold fluorescein signal measured compared to non-electroporated, but otherwise equally treated reference embryos. For extension to other small molecules, the embryos were incubated with 10 ng/ml rhodamine B (**B**-**D**) and 1 μg/ml acridine orange (**E**-**G**) at stage 2a (**B**,**E**), stage 17 (**C**,**F**) and stage 36 (**D**,**G**) for 40 minutes at 27°C. Electroporation was performed using 330 Hz; 15 V; 5000 ms; 1 pulse. The dotted lines demarcate the outlines of: (**B**,**E**) yolk and blastoderm, and (**C**,**D**,**F**,**G**) yolk and embryo. Embryos are shown in lateral view. Scale bar 250 μM.

The electroporation experiments presented so far had been performed with modulation frequencies of 35 kHz. We next varied this frequency and found considerable differences in signal intensity. The optimum for fluorescein uptake appeared at 330 Hz (Figure 6A). In order to extend the results to other small molecules, we selected two additional dyes (rhodamine B and acridine orange) which differ in their molecular properties from fluorescein. Thus rhodamine B becomes strongly enriched in the yolk (Figure 6B-D). Contrary to most other dyes we tested, washing only partly removed the dye from the egg, where it remained trapped in the yolk. Acridine orange showed a more uniform distribution being present both in the yolk and the embryo (Figure 6E-G). At later stages, strong fluorescence was observed in the gallbladder/liver (Figure 6G). It therefore exhibits properties in-between fluorescein and rhodamine B. As for fluorescein, we performed electroporation experiments with rhodamine B and acridine orange. Compared to diffusion alone electroporated embryos always showed an extended uptake of the molecules, demonstrating the potential of electroporation also for other small molecules. Taken together, electroporation enhances the uptake of different small molecules into medaka embryos.

### Lithium induces deficiencies in anterior-posterior development

**Figure 7:**
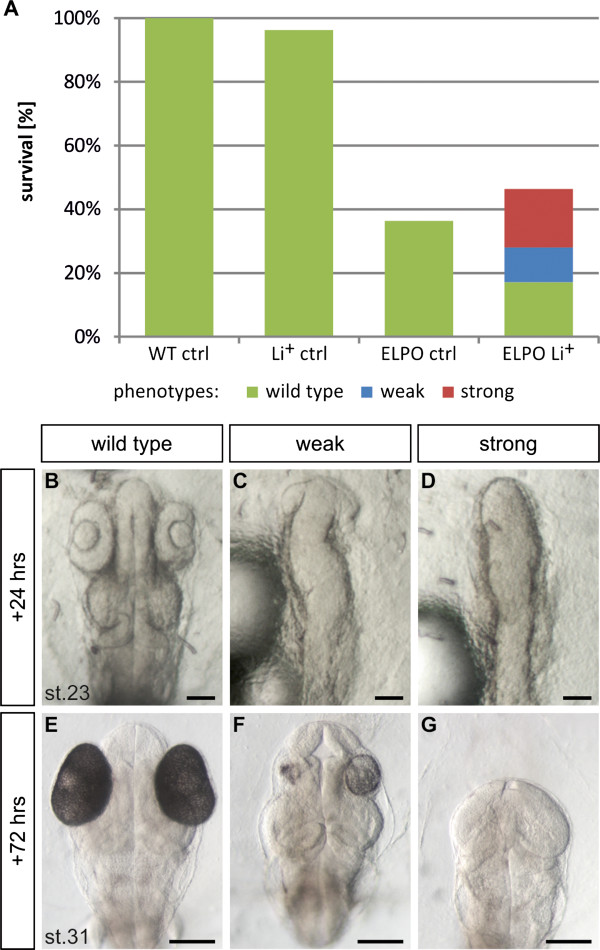
**Lithium induction causes deficiencies in anterior-posterior development.** Embryos at 40% epiboly were exposed to 0.4 M LiCl for 10 minutes at 27°C. Subsequent electroporation was performed at 330 Hz; 15 V; 100 ms; 1 pulse. 24 hours after the induction (+24 hrs) the phenotypes were clearly detectable and finally categorized after 72 hours (+72 hrs) as: wild type (green; **B**, **E**), weak (blue; **C**, **F**) and strong phenotypes (red; **D**, **G**). (**A**) The control (Li^+^ ctrl) shows equally treated but non-electroporated embryos, electroporation control embryos (ELPO ctrl) were electroporated, but not exposed to LiCl. Electroporated embryos with LiCl (ELPO Li^+^) showed phenotypes indicated by colours. Embryos are shown in dorsal view with anterior to the top. Scale bar 100 μM.

After the analysis of the diffusion properties of medaka eggs and the establishment of electroporation as a tool to improve the uptake, we tested the procedure for our initial experiments with lithium chloride. For this we used 0.4 M lithium chloride, a concentration comparable to the one used in zebrafish experiments [13]. Also the incubation time (10 minutes) was in agreement with the zebrafish experiments. Diffusion alone at stage 14 (40% epiboly) resulted in normal development (Figure 7A). However, more than 60% of the embryos that were electroporated (15 V, 330 Hz, 100 ms, 1 pulse) in the presence of lithium chloride showed clear deficiencies in anterior-posterior development (Figure 7A), with 24% developing a weak phenotype and 39% a strong phenotype (Figure 7A and Additional file 5). As a strong phenotype we classified all embryos that showed severe axis truncation anterior to the midbrain, resulting in missing eyes and forebrain (Figure 7D,G), whereas in weak phenotypes only the eyes were affected (Figure 7C,F). Furthermore, we observed the formation of enlarged and ectopic otic vesicles (Additional file 6), which again is in good agreement with the phenotypes seen for *wnt1* overexpression [17]. Control embryos that were electroporated and not treated with lithium chloride developed normally, but showed an elevated mortality rate (Figure 7A and Additional file 5). Nevertheless, electroporation could be established as a tool to enhance the uptake of small molecules into medaka embryos in order to study effects on development.

## Discussion

Several studies have been performed to qualify the effect of small molecules on fish embryos [21-23]. In addition, toxicological applications like the FET test are discussed as an alternative to experiments with mammals and birds [8]. The uptake of substances by the embryos has been recognized as a critical factor in these experiments [24,25]. However, little is known about the diffusion properties of the chorion and other membrane systems covering fish embryos. We used fluorescing substances as a model to study the diffusion of small molecules into medaka embryos. Combined with fluorescence microscopy quantification this offers a number of advantages over conventional techniques like radioactive labelling. Firstly, individual embryos can be followed over the time and quantified repeatedly. Secondly, the internal distribution of the molecules in the embryo due to the physicochemical nature of the molecules can be examined and compared at different stages of development. Alterations in the compartment specific distribution, as for example after incubation with detergents (Figure 3), are highly important for the assessment of pharmacokinetic properties of drugs. Thirdly, metabolic processing can be followed (e.g. as for the accumulation of fluorescein and acridine orange in the liver/gallbladder). And finally, the distribution in different compartments due to variable diffusion rates can be detected, as the accumulation in the perivitelline space (Figure 2F-H”).

We used fluorescein, rhodamine B, acridine orange, and lithium chloride for our experiments. They represent small molecules which cover a large spectrum of different properties from charged (lithium chloride) to non-charged (acridine orange). The substances exhibited varying distributions in medaka eggs, indicating differences in their hydrophobic properties (rhodamine B being most strongly enriched in the yolk). Nevertheless, all substances showed low diffusion rates into medaka embryos. In contrast to zebrafish, medaka eggs contain a hard chorion. Based on suggestions from the literature [3], we therefore suspected the chorion to represent a diffusion barrier, shielding the embryos from their chemical environment. Following sperm entry, a calcium wave moves along the surface and the chorion matures [26], thereby reaching maximum hardness approximately 6 hours after fertilization [12]. We therefore expected a dramatic reduction of the diffusion rate after chorion hardening. However, no differences appeared in the experiments (Figure 1E). As the chorion permeability is connected to calcium ions [27,28] we incubated the embryos right after fertilization in a calcium-free medium [29], with different pH values and even tried incubation in distilled water. However, the uptake of fluorescein into the embryos did not improve (data not shown). Therefore, conditions affecting chorion hardening did not alter the diffusion rates into the egg, neither did dechorionation of the embryos (Figure 2D’, E). Instead we observed a rapid diffusion through the chorion into dead embryos (Figure 2C’.E). The fact that inner membrane systems and not the chorion represent the main diffusion barrier in medaka eggs was further supported by the fast accumulation of signals in the perivitelline space (early in-diffusion; Figure 2F-F”) and the rapid loss of this signal during out-diffusion (Figure 2G-H”). On the contrary, accumulation in the embryo needed extensive in-diffusion times, but once achieved stayed considerably longer than that of the perivitelline space (Figure 2F-H”). The result is a bi-phasic progression of diffusion (Figure 2A), caused by a strong diffusion barrier positioned closer to the embryo than the chorion. During blastula stage, the egg can be subdivided into three distinct cell lineages. One lineage consists of the pluripotent deep layer blastomeres (DEL), which produce the future embryo proper. The second domain consists of a syncytium of multiple nuclei, called the yolk syncytial layer (YSL). The third domain is the envelope layer (EVL), a thin cell layer which covers the DEL and will eventually form the periderm [30]. Both the second and the third domain are extra-embryonic. According to our results, the EVL/periderm would be a candidate to block the diffusion into the egg.

These results have important implications for pharmacologic and toxicologic experiments with medaka embryos. Due to the bi-phasic diffusion process, the substance is initially enriched in the perivitelline space (diffusion half-time in the range of a few minutes). Subsequently, the slow diffusion into the embryo/yolk starts (diffusion half-time in the range of several hours). Therefore extended exposure to the test substances is necessary to evaluate pharmacologic/toxicologic consequences on embryonic development. Due to the slow accumulation in the embryo, effects on the first hours of development (e.g. early teratogenic properties) will not be detectable without enhanced transfer.

Assuming that the main diffusion barrier is a membrane system, it should be possible to increase its permeability by addition of detergents. Indeed, addition of Triton X-100 improved the uptake of fluorescein dramatically, but severely affected the membrane systems of the embryo (see Figure 3). Also injection into the yolk turned out to be inefficient. A possibility to make membranes permeable is electroporation. We applied current shifted radio frequency pulses [20] and could optimize the conditions to substantially increase the dye uptake compared to diffusion alone. Also other electroporation techniques like nanosecond pulsed electric fields have been tested for this purpose [31]. Such techniques would not only be of interest for small molecules, but also for the transfer of DNA or RNA. Indeed, Hostetler and colleagues initially proposed this technique to obtain transgenic medaka fish. In our hand the method worked effectively for small molecules, however, we failed with DNA. Closer inspection revealed that not even fluorescence labelled oligonucleotides (MW 6000) could pass the chorion, suggesting a size exclusion for the pores of the chorion in that range (data not shown). Electroporation did not improve this transfer. We also used DNA expression constructs containing gfp and luciferase for more sensitive assays, but failed to detect any marker gene expression in intact electroporated embryos. Only after dechorionation we could successfully transfer DNA into the embryos by electroporation (detection of gfp expression; data not shown). However, the survival of dechorionated embryos is extremely low during electroporation, making the method inapplicable for routine experiments. Injection of DNA into the perivitelline space and subsequent electroporation would be an option. However, direct injection of DNA into the zygote represents a simple alternative.

Having established electroporation for the uptake of small molecules into the living medaka embryo, we then returned to our initial question, the application of lithium to medaka development. The effects of lithium on developmental processes have been shown in several organisms. Already more than 60 years ago it was known that amphibian embryos developed severe anterior truncations when exposed to lithium during gastrulation (reviewed in [18]). This dorsalization effect was explained by the finding that lithium inhibits GSK-3β, a key component of the Wnt signalling pathway [32,33]. Indeed, diffusion and subsequent electroporation led to axis truncations in medaka embryos, similar to those observed upon ectopic expression of *wnt1*[17]. The embryos either failed to develop anteriorly to the midbrain (Figure 7D,G), lacking eyes and the forebrain (strong phenotype), or they developed eyes with reduced size (Figure 7C,F; weak phenotype). Electroporation therefore effectively improves the transfer of small molecules into medaka embryos. Hence, this method can be used to enhance the uptake of chemicals. Examples of such applications are specific inducers or repressors of signalling pathways to study embryonic development. For these experiments timing represents an important parameter. Slow diffusion and consequently slow accumulation prevents an exact timing of the effective concentration in the embryo. Electroporation strongly increases the internal concentration within a single step as exemplified for lithium chloride (the timing of Wnt signalling pathway activation critically affects the observed phenotypes; [17]).

## Conclusion

During our experiments we found that membrane layers surrounding the medaka embryo represent a diffusion barrier for small molecules, whereas the hard outer chorion is readily passed. The slow diffusion has to be considered when toxicologic or pharmacologic experiments are performed with medaka embryos. A possible way to overcome this problem is electroporation, which substantially improves the uptake of small molecules. We thus were able to induce medaka embryos with the GSK-3 inhibitor lithium and could show that the resulting activation of the Wnt signalling pathway causes deficiencies in anterior-posterior development.

## Competing interests

The authors declare that they have no competing interests.

## Authors’ contributions

GJ carried out the majority of diffusion experiments, the electroporation experiments with lithium and drafted the manuscript. MH performed all other electroporation experiments. AF participated in the diffusion experiments. CH and JW build the electroporation apparatus. TC was responsible for coordination of the experiments and writing of the manuscript. All authors read and approved the final manuscript.

## Supplementary Material

Additional file 1 **Fluorescein enrichment in the gallbladder/liver.** Embryos at the 1-cell stage were incubated with 10 mg/ml fluorescein for 40 minutes at 27°C. Standard washing steps were performed and pictures were taken after 3 days (stage 31). Embryos are shown in lateral view with anterior to the left. (A) Fluorescence and (B) bright field pictures of the eggs, arrow heads indicate staining in the gallbladder. Scale bar 250 μM. Click here for file

Additional file 2 **Methylene blue diffusion into embryos. Embryos at the 1-cell stage were incubated with 0.001% methylene blue in 10× ERM for 10 minutes at 27°C.** Standard washing steps were performed and brightfield pictures were taken after 10 (A) and 20 minutes (B) of washing. Embryos are shown in lateral view; the blastomeres are on the top. Scale bar 250 μM. Abbreviations: DIFF, diffusion. Click here for file

Additional file 3 **Yolk injection.** Embryos at the 1-cell stage (upper row) and stage 30 (lower row) were injected with 10 mg/ml fluorescein. Embryos are shown in lateral view, blastomeres (A-D) or anterior (E-J) to the top right. The yolk and embryo are demarcated by the dotted lines, the outlines of the chorion by continuous white lines. Incubation time after yolk-injection is indicated by the time designation at the bottom left. In both early (D) and late (I) yolk-injected embryos weak fluorescence (D; arrow head) was only detected after 4 hours within the embryo. Older embryos showed clear enrichment of fluorescein within the gallbladder/liver (I,J; arrow). Scale bar 250 μM. Abbreviations: min, minutes; hrs, hours. Click here for file

Additional file 4 **Results of electroporation optimization experiments.** Embryos at stage 17 were incubated with 10 μg/ml fluorescein for 40 minutes at 27°C. Subsequent electroporation was performed at 15 kHz with varying voltages and pulse lengths. Fluorescence intensity is shown in the top row and was normalized to the values measured for the diffusion control. Survival: percentage of surviving embryos after 20 minutes of washing. In order to obtain more significant results, experiments with similar conditions (voltage) were combined. Click here for file

Additional file 5 **Electroporation of lithium incubated embryos.** Embryos at 40% epiboly were incubated with 0.4 μM lithium chloride for 10 minutes at 27°C. Thereafter, embryos were either directly transferred into ERM (Diffusion), or electroporation was performed at 330 Hz, 15 V, 100 ms using a single pulse (Electroporation). Phenotypes were categorized into strong (eyes were missing) and weak (only small eyes developed) 72 hours after induction. ^*)^ Ectopic otic vesicles were observed only in embryos developing a strong phenotype and were not considered in the calculation of the percentage of phenotypes in surviving embryos. Click here for file

Additional file 6 **Ectopic otic vesicles in lithium induced embryos.** Embryos at 40% epiboly were exposed to 0.4 M LiCl for 10 minutes at 27°C followed by electroporation at 330 Hz; 15 V; 100 ms; 1 pulse (longer pulse lengths resulted in reduced survival of the embryos). Embryo is shown in dorsal view with anterior to the top 72 hours after the induction, black arrowheads indicate ectopic otic vesicles. Scale bar 100 μM. Click here for file
