# Supplementary figures and images for: Diffusion of small molecules into medaka embryos improved by electroporation
Source: BMC Biotechnol. 2013 Jul 1;13:53. doi: 10.1186/1472-6750-13-53 (PMC3716799; doi:10.1186/1472-6750-13-53)

**Additional file 1. Fluorescein enrichment in the  
gallbladder/liver**

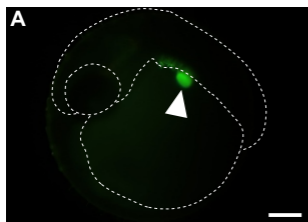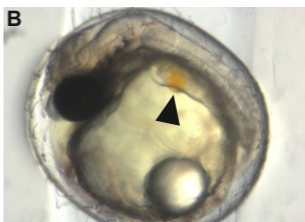

Supplement: Additional file 1 — Fluorescein enrichment in the gallbladder/liver. Embryos at the 1-cell stage were incubated with 10 mg/ml fluorescein for 40 minutes at 27°C. Standard washing steps were performed and pictures were taken after 3 days (stage 31). Embryos are shown in lateral view with anterior to the left. (A) Fluorescence and (B) bright field pictures of the eggs, arrow heads indicate staining in the gallbladder. Scale bar 250 μM. [file 1472-6750-13-53-S1.pdf]

**Additional file 2. Methylene blue diffusion into embryos**

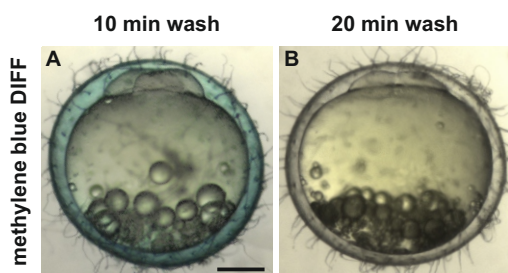

Supplement: Additional file 2 — Methylene blue diffusion into embryos. Embryos at the 1-cell stage were incubated with 0.001% methylene blue in 10× ERM for 10 minutes at 27°C. Standard washing steps were performed and brightfield pictures were taken after 10 (A) and 20 minutes (B) of washing. Embryos are shown in lateral view; the blastomeres are on the top. Scale bar 250 μM. Abbreviations: DIFF, diffusion. [file 1472-6750-13-53-S2.pdf]

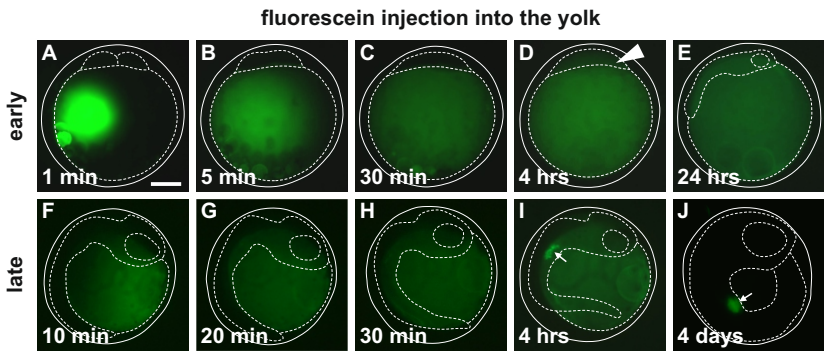

Supplement: Additional file 3 — Yolk injection. Embryos at the 1-cell stage (upper row) and stage 30 (lower row) were injected with 10 mg/ml fluorescein. Embryos are shown in lateral view, blastomeres (A-D) or anterior (E-J) to the top right. The yolk and embryo are demarcated by the dotted lines, the outlines of the chorion by continuous white lines. Incubation time after yolk-injection is indicated by the time designation at the bottom left. In both early (D) and late (I) yolk-injected embryos weak fluorescence (D; arrow head) was only detected after 4 hours within the embryo. Older embryos showed clear enrichment of fluorescein within the gallbladder/liver (I,J; arrow). Scale bar 250 μM. Abbreviations: min, minutes; hrs, hours. [file 1472-6750-13-53-S3.pdf]

**Additional file 6. Ectopic otic vesicles in lithium induced embryos**

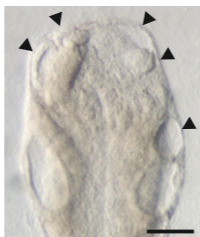

Supplement: Additional file 6 — Ectopic otic vesicles in lithium induced embryos. Embryos at 40% epiboly were exposed to 0.4 M LiCl for 10 minutes at 27°C followed by electroporation at 330 Hz; 15 V; 100 ms; 1 pulse (longer pulse lengths resulted in reduced survival of the embryos). Embryo is shown in dorsal view with anterior to the top 72 hours after the induction, black arrowheads indicate ectopic otic vesicles. Scale bar 100 μM. [file 1472-6750-13-53-S6.pdf]
