# Supplementary material for: Diffusion of small molecules into medaka embryos improved by electroporation
Source: BMC Biotechnol. 2013 Jul 1;13:53. doi: 10.1186/1472-6750-13-53 (PMC3716799; doi:10.1186/1472-6750-13-53)
Supplement: Additional file 4 — Results of electroporation optimization experiments. Embryos at stage 17 were incubated with 10 μg/ml fluorescein for 40 minutes at 27°C. Subsequent electroporation was performed at 15 kHz with varying voltages and pulse lengths. Fluorescence intensity is shown in the top row and was normalized to the values measured for the diffusion control. Survival: percentage of surviving embryos after 20 minutes of washing. In order to obtain more significant results, experiments with similar conditions (voltage) were combined. [file 1472-6750-13-53-S4.pdf]

Additional file 4. Results of electroporation optimization experiments.

	Burst duration [ms]<br>Voltage [V]	15	20	60	150	300	500	1,000	1,500	2,000	5,000
**Relative fluorescence**	**5-10**								3		4
	**15-20**							8	11	21	29
	**25-30**				6	13	14				
	**35-40**			6	18	29					
	**45-50**	5	23	10							
**Survival**	**5-10**								88%		92%
	**15-20**								50%		80%
	**25-30**				83%	82%	100%				
	**35-40**			83%	100%	33%					
	**45-50**	98%	50%	75%		33%					
