# Supplementary material for: Diffusion of small molecules into medaka embryos improved by electroporation
Source: BMC Biotechnol. 2013 Jul 1;13:53. doi: 10.1186/1472-6750-13-53 (PMC3716799; doi:10.1186/1472-6750-13-53)
Supplement: Additional file 5 — Electroporation of lithium incubated embryos. Embryos at 40% epiboly were incubated with 0.4 μM lithium chloride for 10 minutes at 27°C. Thereafter, embryos were either directly transferred into ERM (Diffusion), or electroporation was performed at 330 Hz, 15 V, 100 ms using a single pulse (Electroporation). Phenotypes were categorized into strong (eyes were missing) and weak (only small eyes developed) 72 hours after induction. *) Ectopic otic vesicles were observed only in embryos developing a strong phenotype and were not considered in the calculation of the percentage of phenotypes in surviving embryos. [file 1472-6750-13-53-S5.pdf]

Additional file 5. Electroporation of lithium incubated embryos.

	Diffusion		Electroporation	
**Lithium chloride**	**0 µM**	**0.4 µM**	**0 µM**	**0.4 µM**
Embryos	23	48	36	36
Dead	0	1	12	10
Mortality	0%	2%	33%	28%
Strong phenotype	0	0	0	7
Weak phenotype	0	0	0	7
Ectopic otic vesicles[*)]	0	0	0	4
Normal development	23	47	24	12
**Phenotypes in surviving embryos**	**0%**	**0%**	**0%**	**54%**
